# Cerebrospinal fluid humoral immunity in the differential diagnosis of multiple sclerosis

**DOI:** 10.1371/journal.pone.0181431

**Published:** 2017-07-20

**Authors:** Evanthia Bernitsas, Omar Khan, Sara Razmjou, Alexandros Tselis, Fen Bao, Christina Caon, Scott Millis, Navid Seraji-Bozorgzad

**Affiliations:** 1 Department of Neurology, Wayne State School of Medicine, Detroit, MI, United States of America; 2 Department of Physical Medicine and Rehabilitation; Wayne State University School of Medicine, Detroit, MI, United States of America; Istanbul University, TURKEY

## Abstract

**Background:**

The diagnostic accuracy of cerebrospinal fluid oligoclonal bands (CSF-OCB) detected by isoelectric focusing (IEF) in patients with multiple sclerosis (MS) was evaluated in our study.

**Methods:**

Three hundred and twenty-one patients with MS and other central nervous system (CNS) immune mediated disorders were assessed (CIMD). Cerebrospinal fluid and matched serum samples were examined for the presence of OCB by IEF-IB (isoelectric focusing with immunoblotting).

**Results:**

Isolated oligoclonal bands (ISO-OCB) were the only predictor of MS diagnosis independent of age, gender and CSF-OCB. ISO-OCB ≥ 3.5 detected by IEF yielded a sensitivity of 98% and specificity of 87% in distinguishing MS from MS mimickers.

**Conclusions:**

For the neurologist, a score of ≥ 4 ISO-OCB supports the diagnosis of MS. On the other hand, ISO-OCB ≤3 favors CIMD. Further studies with larger population samples are warranted to confirm these findings.

## Introduction

Multiple Sclerosis (MS) is an inflammatory, demyelinating disease of the central nervous system (CNS) that is most commonly diagnosed between 20 and 40 years of age. Central nervous system (CNS) inflammation in MS is associated with intrathecal production of immunoglobulins (IgG) derived from plasma cells, which represent a restricted set of B cell clones and confirmed by the presence of oligoclonal bands (OCB) in the cerebrospinal fluid (CSF) [1]. The Diagnosis of MS is based on the McDonald criteria entailing dissemination in time and space as demonstrated by clinical and magnetic resonance imaging (MRI) findings [2]. Although CSF-OCB is present in approximately 90–95% of MS patients, they are not unique to MS [3]. Oligoclonal bands have been reported in other primary and secondary CNS immune-mediated disorders (CIMD) that may clinically mimic MS such as CNS lupus, various forms of CNS vasculitis, neurosarcoidosis, antiphospholipid syndrome, CNS infections, CNS lymphoma and neuromyelitis optica spectrum disorder (NMOSD) [3]. However, due to its high diagnostic sensitivity in MS, as well as its high specificity in the appropriate clinical setting, examination of CSF-OCB particularly in the evaluation of patients with early or atypical MS is strongly recommended [4]. Moreover, CSF-OCB positive patients have been associated with a more rapid conversion from clinically isolated syndrome (CIS) to clinically definite MS [5]. Nevertheless, abnormal CSF analysis is not required to establish the diagnosis of clinically definite MS based on McDonald criteria, though the expert panel reaffirmed the significance of abnormal CSF findings in supporting the diagnosis of MS and evaluating alternate diagnosis [2]. A variety of experimental techniques have been used to detect the presence of OCB in the CSF, including agarose gel electrophoresis (AGE) and isoelectric focusing combined with immunoblotting (IEF-IB). Isoelectric focusing combined with immunoblotting requires smaller CSF volume and is more sensitive due to higher resolution peaks, which are easily discriminated from the background [6–8]. The use of IEF-IB has been widely used in the U.S over the past decade. This technique has improved the accuracy in detecting OCB and has shown a robust correlation between OCB and the diagnosis of multiple sclerosis [1]. However, very few studies have examined the utility of CSF IEF-IB in distinguishing MS from other CIMD. We conducted a study to assess the diagnostic capability of CSF-OCB detected by IEF-IB in differentiating MS from other CIMD.

## Patients and methods

### Patients

This study has been approved by the Wayne State Institutional Review Board (IRB# 016612MP4E). A written informed consent has been obtained by all participants in this study. All data was accessed anonymously.

This was a retrospective single center study of patients seen in our Neuroimmunology clinic, for the evaluation of MS or CIMD disorder. Three hundred and twenty-one consecutive patients who underwent CSF analysis between 2011 and 2014 were divided into two groups of MS and CIMD. The MS group met the 2010 McDonald MS criteria (Table 1). The CIDM group included patients with neurosarcoidosis, primary CNS angiitis (PACNS), primary Sjogren’s syndrome, CNS lupus, and antiphospholipid syndrome (Table 1). All CIMD patients met the diagnostic criteria for their respective diagnoses. Central nervous system lupus erythematosus was diagnosed based on the American College of Rheumatology Nomenclature for neuropsychiatric syndromes of systemic lupus erythematosus (ACR AD HOC Committee on Neuropsychiatric Lupus Nomenclature) [9]. Antiphospholipid syndrome (APL) was diagnosed based on the revised international criteria for definite antiphospholipid syndrome [10]. The diagnosis of CNS sarcoidosis, primary CNS angiitis was confirmed by positive biopsy [11, 12].

**Table 1:** Multiple sclerosis (MS) & central nervous system immune-mediated disorder (CIMD) groups.

	Pathology	Number of patients	Number of patients with detected CSF OCB	Race
MS	RRMS	203	198	69 AA/134 W
	PPMS	23	21	7 AA/16 W
	Total	226	219	76 AA/150 W
CIMD	Neurosarcoidosis	52	35	37 AA/15 W
	SLE	18	10	9 AA/ 9 W
	PACNS	6	3	6 W
	Sjogren’s syndrome	14	9	3 AA/11 W
	APL	5	3	2 AA/3 W
	Total	95	60	51 AA/44 W

AA: African-American; W:Whites; RR-MS: Relapsing remitting multiple sclerosis; PP-MS: Primary progressive multiple sclerosis; SLE: systemic lupus erythematosus; APL: antiphospholipid syndrome, PACNS: primary CNS angiitis. CSF-OB were present in 96.9% of MS patients and 63.1% of CIMD patients.

Primary Sjogren’s syndrome was diagnosed based on the criteria established by the American-European Consensus Group and confirmed by biopsy [13]. The clinical history, diagnostic tests and medical records were reviewed for each patient including age, diagnosis and the number of OCB in the CSF cerebrospinal fluid and serum. The mean time interval between the initial symptom and lumbar puncture for the CIMD patients was 8 months (range 1–14 months). Two patients, one with neurosarcoidosis and one with PACNS received treatment with intravenous corticosteroids within 30 days prior to lumbar puncture. Three patients on the CIMD group were on immunosuppressive therapy at the time of the lumbar puncture. Thirty-seven out of 52 patients with neurosarcoidosis (71%) had CNS involvement as first manifestation at the time on their initial assessment and fifteen (29%) had sarcoidosis that previously affected another organ. All patients with SLE and Sjogren’s had systemic involvement.

### Oligoclonal band detection

Cerebrospinal fluid samples were obtained by lumbar puncture that was performed as part of the clinical diagnostic investigations. Matched CSF and serum samples were sent to the hospital laboratory immediately after obtaining the samples for OCB detection.

The method used for detecting OCB was IEF-IB, which is considered the “gold standard" method for OCB detection [6, 14]. In this assay a commercially available FDA-approved kit, produced by Helena Laboratories, Beaumont, TX, was used.

In our laboratory, intrathecal synthesis of IgG was defined as two or more bands present in the CSF but not matched in the serum. CSF-OCB pattern classification was based on two consensus reports recommendation [8, 15]. Five types of patterns were proposed with only patterns 2 and 3 represent intrathecal synthesis of IgG: type 1: normal CSF, type 2: two or more CSF restricted OCB, type 3: CSF restricted OCB and additional, identical OCB in serum and CSF, type 4: identical OCB in CSF and serum, “mirror pattern”, type 5: monoclonal bands in CSF and serum. A “mirror pattern” represents systemic immune reaction with passive transfer of oligoclonal bands from the serum to the CSF through an abnormal blood brain barrier and does not indicate local IgG synthesis. Isolated oligoclonal bands were calculated by subtracting the number of serum OCB from CSF-OCB (CSF OCB minus Serum OCB).

### Statistical analysis

In the univariate analysis, the Mann-Whitney-Wilcoxon test and chi-square test were used to compare the two groups on selected demographic and disease-related variables. Multivariate logistic regression analysis was used to assess the predictive accuracy of ISO-OCB in MS diagnosis, after correction for age, gender and CSF-OCB. Model discrimination was assessed using receiver- operating characteristic, area under curve (AUC, c-statistic). Hosmer-Lemshow test was used to examine model calibration.

## Results

### CSF-OCB and ISO-OCB

Cerebrospinal fluid data of patients with MS (n = 226) and CIMD (n = 95) are showing in Table 2. The MS group had a mean age of 37.5 years, standard deviation (SD) of 13 (range: 18 to 64 years) and the CIMD group had a mean age of 39.2 years, SD of 12.5 (range: 19 to 65 years). The median number of CSF-OCB and serum OCB were 9 (range 3–24) and 0 (range:0–4) in MS group versus 4, (range: 0–8) and 0 (range:0–4) in CIMD group, respectively. The median number of ISO-OCB detected in the MS group was 8 (range 3–19) whereas in CIMD group was 1 (range: 0–6) (Table 2). There were no group differences regarding age (P = 0.65), gender (P = 0.33) or serum OCB (P = 0.80). However, there were significant group differences in the number of CSF-OCB (P<0.001) and ISO-OCB (P<0.001).

**Table 2:** Oligoclonal bands detected by IEF-IB in MS & CIMD patients.

	MS group (n = 226)		CIMD group (n = 95)		P-value
	Median	Range	Median	Range	
CSF OCB	9	3–24	4	0–8	<0.0001
Serum OCB	0	0–3	0	0–4	NS
ISO OCB	8	3–19	1	0–6	<0.0001

IEF: isoelectric focusing; MS: multiple sclerosis; CIMD: central nervous system immune-mediated disorders; NS: not significant; CSFOCB; cerebrospinal fluid oligoclonal bands, ISO-OCB: isolated oligoclonal band

To examine these differences further, we fit a multivariable logistic regression model that included age, gender, CSF-OCB and ISO-OCB. ISO-OCB was the only statistically significant predictor of MS in this model (P<0.05) with an odds ratio of 6.6. Of note, CSF-OCB was not a significant predictor. We then wanted to compare this four-variable model with the single-variable ISO-OCB alone. The likelihood ratio test failed to find a significant difference between four variable models versus the ISO-OCB model (chi square = 3.34, P = 0.32). In addition, the Bayesian Information criterion (BIC) associated with the ISO-OCB only model was substantially lower than that associated with the four-variable model, which indicated superior fit for the simpler model.

**Fig 1:**
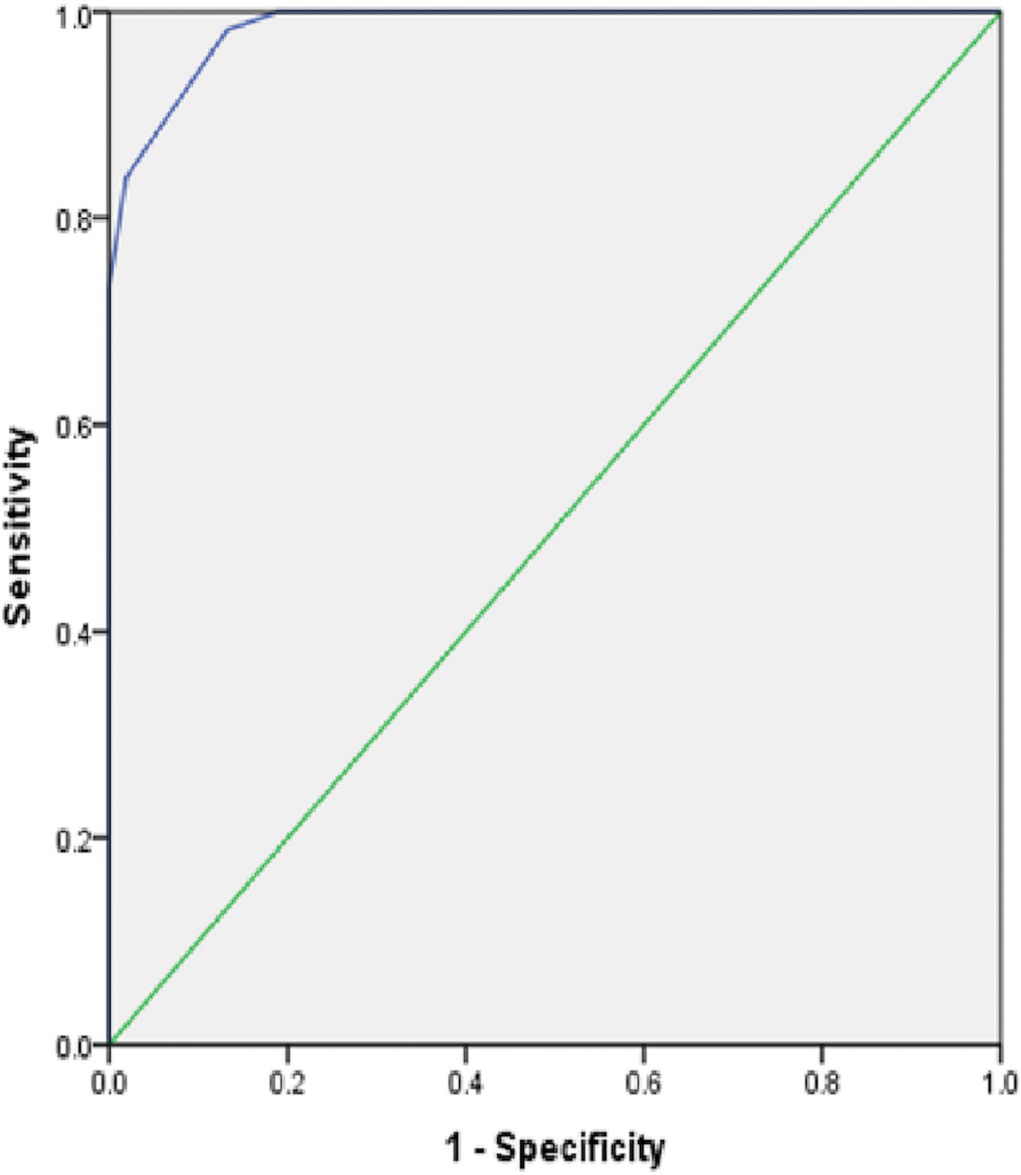
ROC curve plot demonstrating diagnostic accuracy of ISO OCB in MS diagnosis.

A receiver operating characteristics (ROC) curve analysis was carried out to evaluate the diagnostic accuracy of using ISO-OCB alone in predicting MS diagnosis. Diagnostic discriminatory power between MS and non- MS groups was remarkable as indicated by the area under the curve (AUC) of 98.5% (Fig 1). The Hosmler-Lemshow test indicated that the model was well calibrated (P = 0.99). We used Youden’s method to select an optimal cut-point for ISO-OCB in predicting MS. The Youden method maximized the sum of sensitivity and specificity. Isolated oligoclonal bands greater than or equal to 3.5 have a sensitivity of 0.98, specificity of 0.87.

### CSF OCB patterns

Table 3 presents the CSF-OCB patterns in MS and CIMD patients. Among the 226 patients with MS, seven patients (3.1%) showed pattern 1 with no intrathecal IgG synthesis, 202 (88.5%) had pattern 2 with OCB restricted to the CSF and 17 (8.4%) had pattern 3, with CSF restricted OCB and additional identical bands in CSF and serum. Overall, 96.9% of the MS patients had intrathecal IgG synthesis.

**Table 3:** CSF OCB patterns in MS and CIMD groups.

		Pattern 1 No (%)	Pattern 2 No (%)	Pattern 3 No (%)	Pattern 4 No (%)
	RRMS	5 2.5	188 92.6	10 4.9	0
MS	PPMS	2 8.7	12 52.2	9 39.1	0
	Total	7 3.1	200 88.5	19 8.4	0
	Neurosarcoid	15 28.9	19 36.5	16 30.8	2
CIMD	SLE	7 38.9	7 38.9	3 16.6	1
	PACNS	3 50	3 50	0	0
	Sjogren’s syndrome	5 35.7	4 28.6	5 35.7	0
	APL	2 40	1 20	2 40	0
	Total	32 33.7	34 35.8	26 27.3	3 3.1

Among the 95 patients with CIMD, 32 (33.7%) had no specific CSF bands, 34 (35.8%) had CSF restricted OCB and 26 (27.4%) showed pattern 3 with CSF restricted OCB and additional identical bands in CSF and serum. Only three patients (3.1%), including two with neurosarcoidosis and one with SLE showed pattern 4 (“mirror pattern” with equal number of identical bands in the CSF and serum) and were excluded from the analysis. Overall, 63.1% of the CIMD patients had intrathecal synthesis of oligoclonal IgG (Table 3).

## Discussion

The use of IEF-IB method in detecting OCB has been evaluated in the diagnosis of MS. Up to 95% of patients with clinically definite MS are identified with OCB using this sensitive technique [16]. Although IEF-IB is well established as a sensitive laboratory test to detect OCB [3], the diagnostic value of OCB in distinguishing MS patients from CIMD is not well established. In our study, a cut off score of ≥ 3.5 ISO-OCB yielded a sensitivity of 98% and specificity of 87% in differentiating MS from MS mimickers that may also trigger a humoral response with OCB production in the CSF, independent of age, gender and CSF-OCB. However, in practice, since 0.5 band has no practical application, a cut off score of ≥ 4 ISO-OCB favors the diagnosis of MS, while a score of ≤ 3 ISO-OCB supports CIMD.

In a similar study, Fortini et al. [6] suggested a cut off score of ≥ 4 ISO-OCB with a sensitivity of 90% and a specificity of 94% in distinguishing MS from non-MS cases. However, the number of non-MS patients in that study was small and only two were categorized as inflammatory cases in the non-MS group.

Bourahoui et al. studied a larger cohort of MS and other neurological disorders (1292 patients) [17], and found a cut off score of ≥ 3 OCB with a sensitivity of 85% and specificity of 92% for the diagnosis of MS. They also used IEF-IB method for CSF-OCB analysis. However, our study population is different from Bourahoui et al in terms of including both African-American and white patients, as well as our patient population in CIMD group includes more cases of neurosarcoidosis. Additionally, we focus on the diagnostic value of ISO-OCB, whereas Bourahoui et al studied the cut-off score for CSF-OCB.

Mygland et al, [18] found that the number of OCB detected by IEF method was similar in MS and other inflammatory disorders but lower in non-inflammatory disorders leading to the conclusion that a high number of OCB is suggestive of CNS inflammatory diseases but not useful in distinguishing MS from other CIMD.

Cerebrospinal fluid OCB reflects a local plasma cell response in CNS inflammation and the analysis of CSF-OCB plays an important role in the diagnostic process of autoimmune diseases affecting the CNS, particularly MS. Two consensus reports [8,15] recommended the use of IEF-IB to detect CSF-OCB as a routine test to support the diagnosis of MS. Despite these recommendations, the exact cut off value for the diagnosis of MS has not been determined. This is of particular importance when considering infections, paraneoplastic disorders, systemic and CNS inflammatory disorders or any other neurological disorders that can provoke humoral response resulting in CSF-OCB [3]. Notably, OCB have been demonstrated in almost 30–50% of CNS inflammatory conditions as well as 5–10% of non-inflammatory neurological disorders [19]. Thus, determining a sensitive cut off point in differentiating MS from other CIMD and neurological disorders could benefit the clinician. Furthermore, other CSF findings, such as protein, glucose and lactate level, total cell count (including white blood cell count and differential, red blood cell count), gross characteristics, CSF-serum albumin ratio, provide more information and may point to a different etiology rather than MS. A thorough consideration of all components of CSF analysis is a key factor for a correct diagnosis.

In our study, 63.1% of patients with CIMD showed intrathecal OCB synthesis. Our results are somewhat higher than those reported in previous studies [20–22]. A possible explanation is that the CSF analysis was performed relatively early in the disease process and a very small number of patients were treated with steroids or immunosuppressive therapy.

There are several limitations to our study that necessitate careful interpretation of our results. This was a retrospective analysis of the data and ascertainment bias could not be excluded. The diagnostic accuracy of OCB in distinguishing MS from other CIMD remains to be established, but our data indicate that the detection of ISO CSF OCB is worth exploring in larger multi-center cohorts. Due to lack of data for few MS patients, disease duration was not considered for the MS group. Walsh and Tourtelotte [23] showed no alteration in the OCB pattern overtime in the MS population. Axelsson et al [24] showed that the OCB pattern changed during long-term follow-up in MS, but found no relation between disease duration and altered pattern. Although most of the prior studies support the lack of impact of the disease duration on the intrathecal production of CSF-OCB in MS, there is no general consensus [25]. In addition, there is a limited number of studies examining the correlation between the disease duration and the CSF-OCB formation in CIMD [22, 26]. We did not include imaging data to explore the potential correlation with MRI findings such as lesion load or brain volume loss due to modest sample size and lack of standardized MRI scans. Our patient population in the CIMD group was predominantly neurosarcoidosis and although we had different types of CIMD such as CNS vasculitis, Sjogren’s syndrome, SLE and APL, the number of patients in each disease category was not sufficient enough for subgroup analysis. Furthermore, CIMD has a wide spectrum and other types of CIMD such as Behcet's disease, paraneoplastic disorders and acute demyelinating encephalomyelitis that were not assessed in our study. Moreover, we did not include patients with clinically isolated syndrome to validate our findings in that clinical subtype of MS, where an ISO-OCB cut off score could be most valuable. Previous studies suggested that intrathecal OCB synthesis is an independent risk factor for conversion to MS in patients with CIS and doubles the risk of having a second attack but does not predict disease progression [27, 28]. Recent studies confirmed that the risk of conversion from CIS to MS is higher if OCBs are present at onset [29]. This finding is especially significant for patients with CIS and normal MRI scan and can assist in clinical decision making. In a large, multicenter trial, Kuhle et al identified OCB status as a strong and independent predictor of conversion to definite MS [30].

In this study we focused on the diagnostic accuracy of ISO-OCB in differentiating MS from CIMD. Future directions include determining whether the elevated IgG synthesis rate or a high CSF IgG index with absent CSF-OCB, which may occur in the minority of MS cases [3], has similar implications to the ISO-OCB and what the cut off value is. It would be also interesting to examine whether an elevated CSF IgG index may add diagnostic value to a borderline or even lower than 3.5 ISO-OCB. As stated above, an ISO-OCB cut off could be more valuable in an earlier cohort of patients with clinically (CIS) or radiologically isolated syndrome (RIS), where diagnosis can be challenging. Including this cohort in a prospective study would allow further evaluation of the role of ISO-OCB in the differential diagnosis of these diseases and would guide treatment decisions earlier in the disease process.

The revised 2010 McDonald criteria do not require positive CSF findings in order to diagnose MS. The CSF status was not evaluated for its contribution to the MAGNIMS criteria for dissemination in time and space [2]. We hope that this study will contribute to further discussion and improve the diagnostic accuracy in cases that do not completely meet the criteria for the diagnosis of MS.

In conclusion, there is paucity of data regarding the significance of a cut off score for ISO- OCB in the MS diagnostic scheme. Central nervous system immune-mediated disorders may have a clinical course similar to MS and the differential diagnosis could be challenging. Thus, larger studies validating our findings would be of significant value to the clinician in potentially differentiating MS from MS mimickers.

## Supporting information

S1 Report Summary Incidence of CSF OCB in different clinical series. (DOCX)
