# Supplementary material for: Cerebrospinal fluid humoral immunity in the differential diagnosis of multiple sclerosis
Source: PLoS One. 2017 Jul 20;12(7):e0181431. doi: 10.1371/journal.pone.0181431 (PMC5519077; doi:10.1371/journal.pone.0181431)
Supplement: S1 Report Summary — (DOCX) [file pone.0181431.s001.docx]

|  | Neurosarcoid | CNS SLE | Sjogren’s | APL |
| --- | --- | --- | --- | --- |
| Wengert et al (2013) | 22% |  |  |  |
| Joseph et al 2009 | 27% |  |  |  |
| Marangoni et al 2006 | 71.5% (small study) |  |  |  |
| Barahoui et al (2004) | 9% | 16% |  | 33% |
| McLean et al (1995) | 51% | 25% |  |  |
| Zajicek et al (1998) | 37% |  |  |  |
| Scott et al (1989) | 29% |  |  |  |
| Alexander et al (1986) |  |  | 86% (1 or more OCB) |  |
| Alexander et al (1986) 2^nd^ study |  |  | 25% |  |
| Ernerudh et al (1985) |  | 81.8% |  |  |
| Winfield et al (1983) |  | 42% |  |  |
| Seibold et al (1982) |  | 97% |  |  |
| Siltzbach et al (1967) |  | 42% |  |  |
